# Pulmonary thromboembolism after operation for bilateral open distal radius fractures: a case report

**DOI:** 10.1186/1756-0500-7-36

**Published:** 2014-01-14

**Authors:** Yuka Igeta, Kiyohito Naito, Yoichi Sugiyama, Kazuo Kaneko, Osamu Obayashi

**Affiliations:** 1 Department of Orthopaedic Surgery, Juntendo University Shizuoka Hospital, 1129 Nagaoka, Izunokuni, Shizuoka 410-2295, Japan; 2 Department of Orthopaedic Surgery, Juntendo University School of Medicine, 2-1-1 Hongo, Bunkyo, Tokyo 113-8421, Japan

**Keywords:** Distal radius fracture, Depression, Pulmonary thromboembolism, Activity of daily living

## Abstract

**Background:**

Pulmonary thromboembolism after upper extremity operation is rare. We report a patient with thromboembolism after debridement open reduction and internal fixation for bilateral open distal radius fractures.

**Case presentation:**

The Japanese patient was an 80-year-old previously healthy female who was able to walk on her own. She fell down and was taken to our hospital. She was diagnosed with bilateral open distal radius fractures and we performed debridement open reduction and internal fixation on the same day. Although she could not walk and was depressed, she was discharged on the ninth postoperative day. However, on the eleventh postoperative day, she returned to our emergency department with complaints of dyspnea and cold sweat. Her serum D-dimer level was 19.0 μg/dl, troponin T was positive, and urgent contrast computed tomography scan of her thorax revealed thrombosis in the bilateral main pulmonary artery. She was diagnosed with pulmonary thromboembolism and admitted to our hospital again. On the second admission, although she had breathing problems, she did not require a respirator. Oxygen was supplied as well as anticoagulants. On the seventh day after being diagnosed with embolism, thrombosis in the bilateral main pulmonary arteries had disappeared.

**Conclusion:**

The patient did not have any “strong” risk factors as reported in the Japanese Orthopedic Association Clinical Practice Guideline on the Prevention of Venous Thromboembolism in Patients Undergoing Orthopedic Treatments. In general, upper extremity operation carries a low risk for pulmonary thromboembolism. For patients with decreased activity of daily living and depression, we should consider postponing discharge and performing rehabilitation until activity of daily living is improved.

## Background

Pulmonary thromboembolism and deep vein thromboembolism are complications after orthopedic operations. Recently, some methods for prophylaxis have been reported [1-4]. Guidelines have been published for numerous countries and they indicate the importance of taking preventative measures for large joint operations, for example knee or hip joint operations (American College of Chest Physicians Evidence-Based Clinical Practice Guidelines (ACCP) [5]; Japanese Orthopaedic Association (JOA) [6]; and National Institute of Health and Clinical Excellence Guidelines (NICE) [7]). However, there is no consensus of prophylactic treatments for pulmonary thromboembolism after upper extremity operation. For patients who would undergo operations of the upper extremities and would be able to get out of bed and walk soon thereafter, no aggressive prophylactic treatments are recommended [5,6].

In this case report, we present a patient who experienced pulmonary thromboembolism after operation for bilateral open distal radius fractures.

## Case presentation

**Figure 1:**
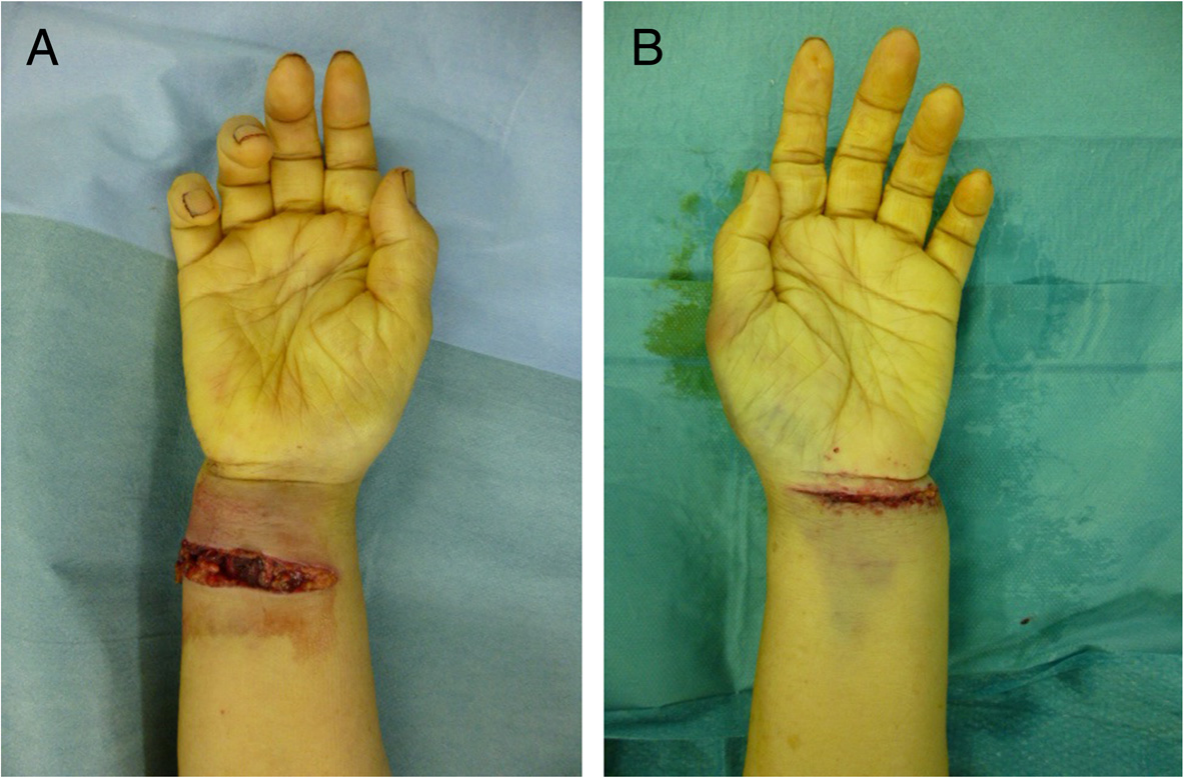
**Open wound on the palmar side of wrists. A**: Right side. **B**: Left side.

**Figure 2:**
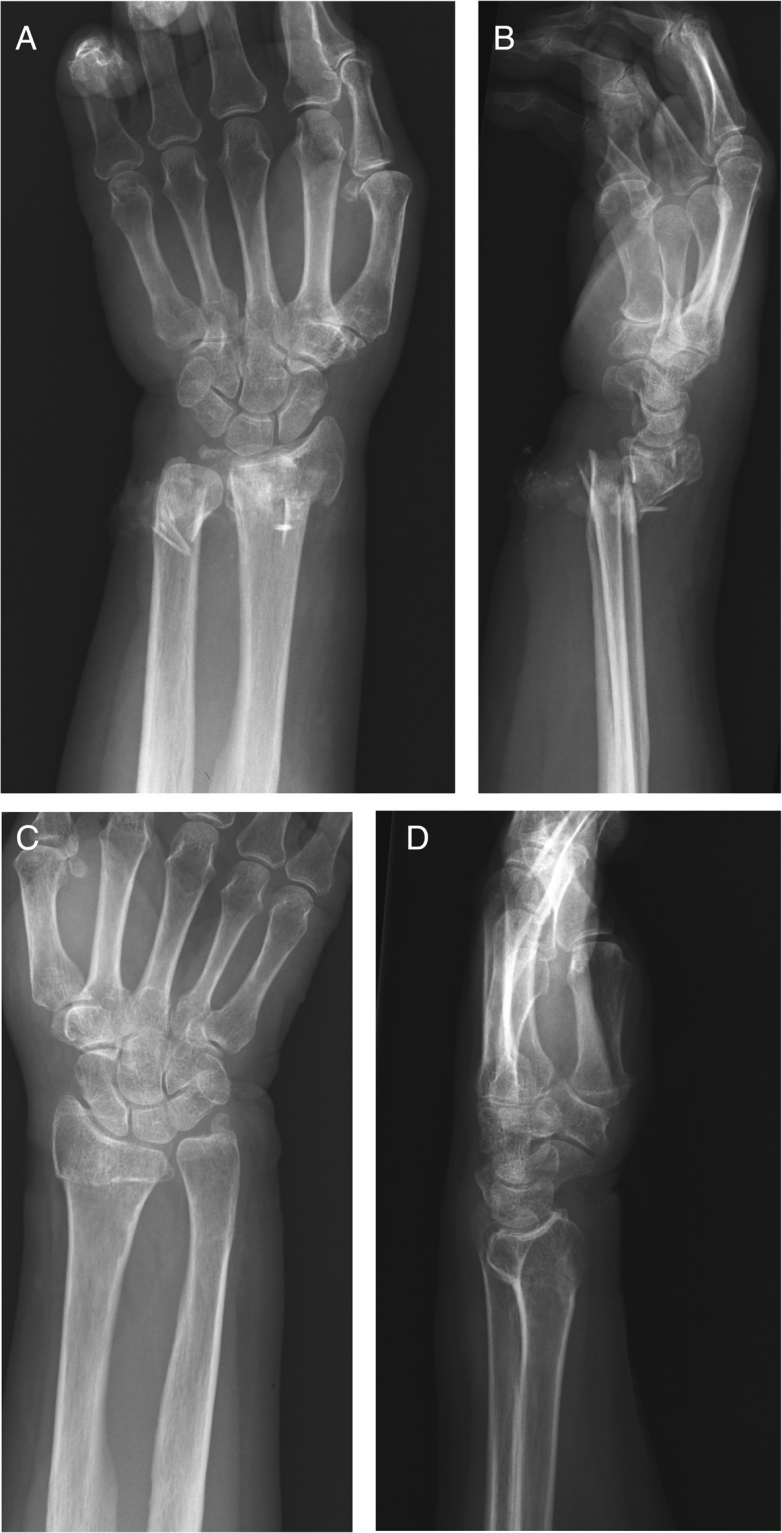
**Radiographs showed bilateral distal radius fractures.** According to the Arbeitsgemeinschaft für Osteosynthesefragen (AO) classification, right side is A3 and left side is A1. **A**: Right side, A-P view. **B**: Right side, lateral view. **C**: Left side, A-P view. **D**: Left side, lateral view.

We report a case of an 80-year-old previously healthy Japanese female patient who was able to walk on her own. She fell down and was taken to our hospital. There were cut wounds measuring approximately 5 cm on the palmar side of both wrists (Figure 1A and B) and radiographs showed bilateral distal radius fractures. The right side was classified as A3 according to the Arbeitsgemeinschaft für Osteosynthesefragen (AO) classification and the left side was A1 (Figure 2A,B,C and D). She was diagnosed with bilateral open distal radius fractures and we performed debridement open reduction and internal fixation on the same day.

**Figure 3:**
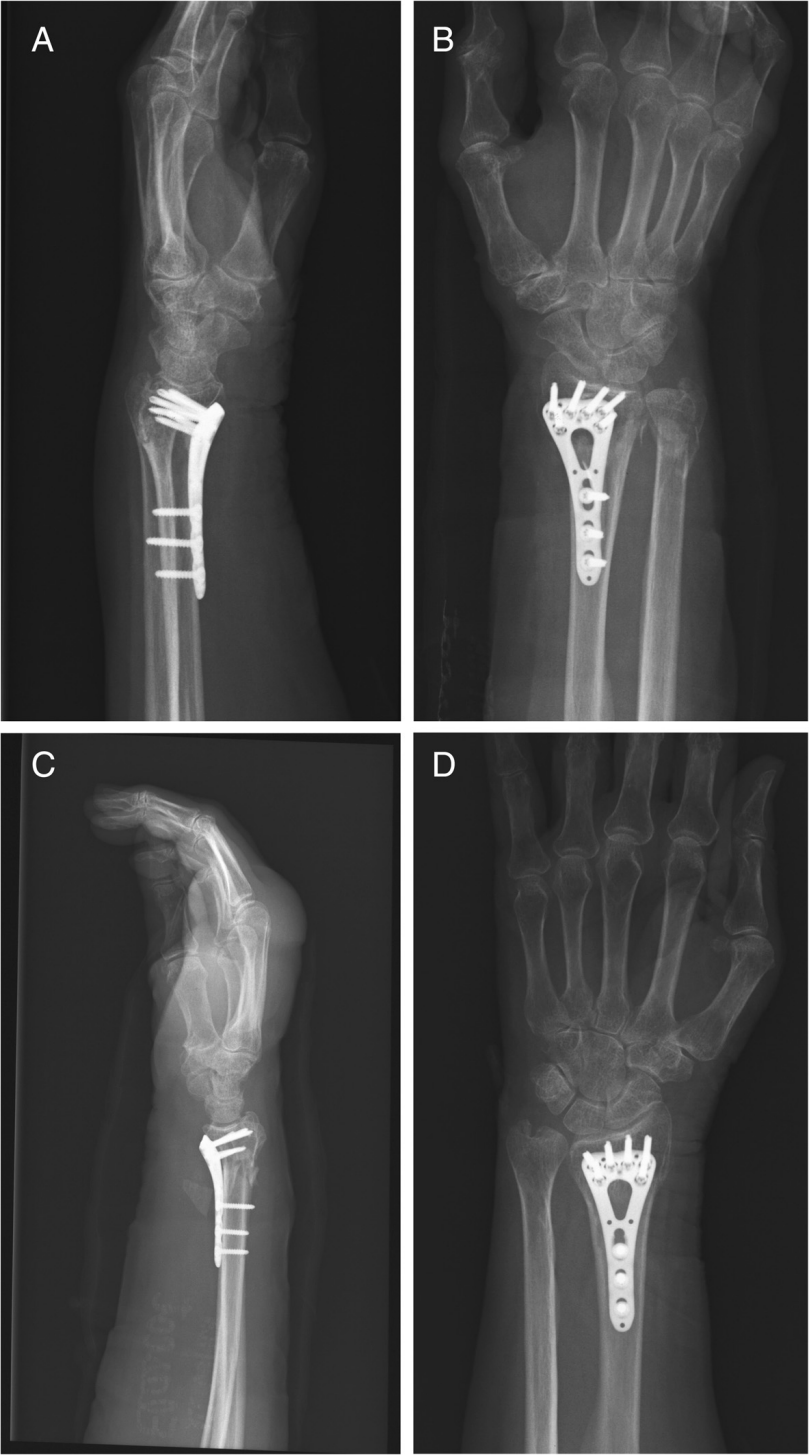
**Postoperative radiographs. A**: Right side, A-P view. **B**: Right side, lateral view. **C**: Left side, A-P view. **D**: Left side, lateral view.

We resected skin of the edges in the wounds and debrided the contaminated tissues in the wounds. Since contamination of bones was almost nonexistent, we performed osteosynthesis with volar locking plates (Figure 3A,B,C and D). No postoperative immobilization was prescribed and the patient was encouraged to use her upper limb immediately after the operation. Also, patient was allowed to get out of bed the day after the surgery. However, she was unwilling to leave her bed due to the three factors; depression, strong pain and her age.

**Figure 4:**
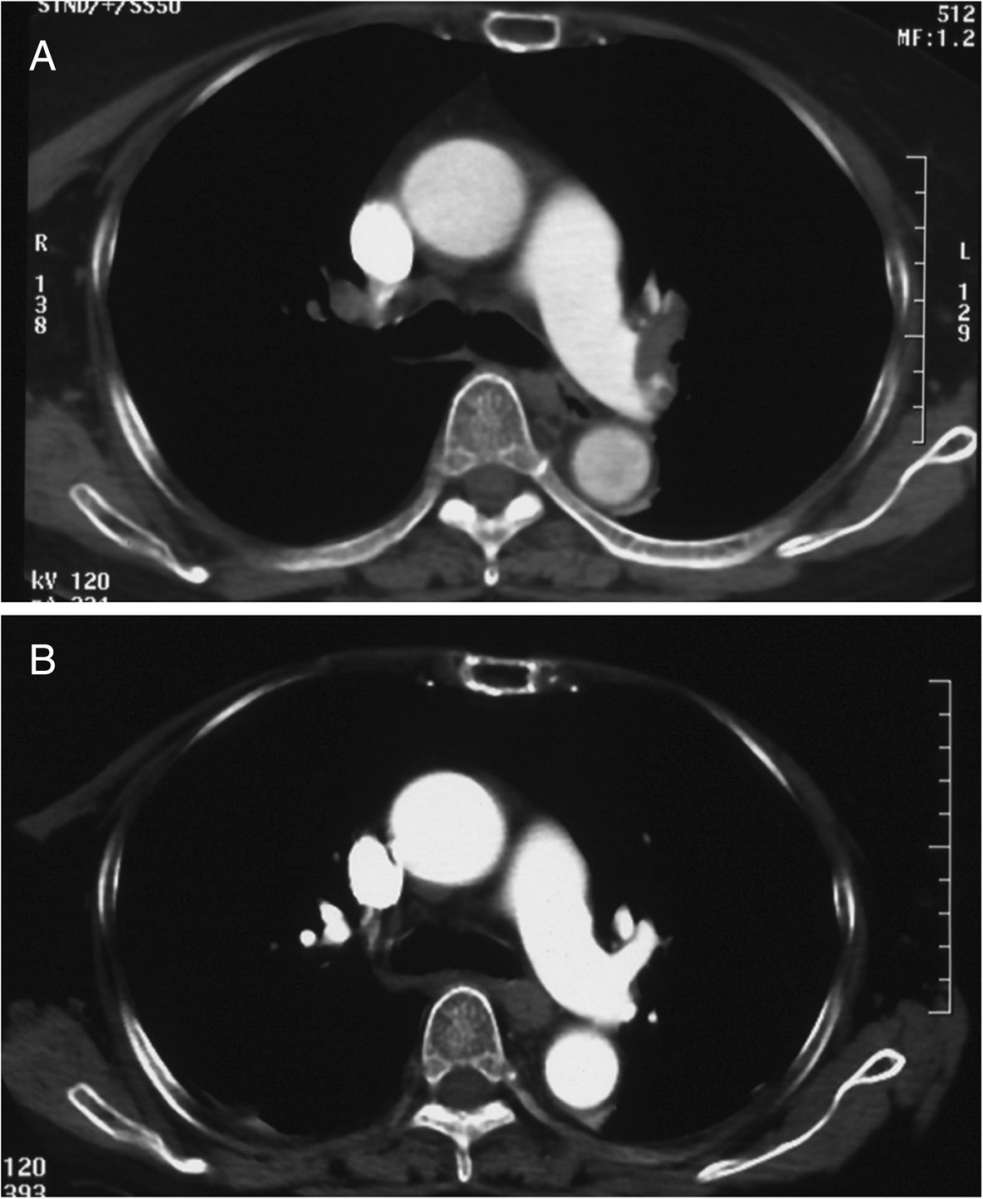
**Pulmonary thromboembolism on computed tomography (CT) scan. A**: CT scan showed thrombosis in the bilateral main pulmonary arteries. **B**: Disappered thrombosis in the bilateral main pulmonary arteries.

On the eleventh postoperative day, she returned to our emergency department with complaints of dyspnea and cold sweat. Her serum D-dimer level was 19.0 μg/dl, troponin T was positive and urgent contrast computed tomography (CT) scan of her thorax revealed thrombosis in her bilateral main pulmonary arteries (Figure 4A). She was diagnosed with pulmonary thromboembolism and was immediately admitted.

Although she had breathing problems, she did not require a respirator. Oxygen was supplied as well as anticoagulants (urokinase, heparin). On the seventh day after the embolism was found, her condition in general became stable, and thrombosis in her bilateral main pulmonary arteries disappeared on CT scan (Figure 4B). She started taking oral administration of warfarin and the intravenous heparin therapy was stopped. Twelve months after the operation, there was no embolism relapsed and she had attained her original activities of daily life.

## Discussion

For patients who would undergo operations of the upper extremities and would be able to get out of bed and walk soon thereafter, no aggressive prophylactic treatment are recommended [5,6]. Also, there are no common prophylactic guidelines for pulmonary thromboembolism after operations on upper extremity. The guidelines in other countries including the United States and the United Kingdom make no references on this issue [5,7].

NICE, which are the guidelines for the United Kingdom, say that the following patients should be treated with anticoagulants: patients with active cancer or undergoing cancer treatment, age over 60 years, critical care admission, dehydration, known thrombophilias, obesity (body mass index over 30 kg/m^2^), one or more significant medical comorbidities (for example: heart disease; metabolic, endocrine or respiratory pathologies; acute infectious diseases; inflammatory conditions), personal history or first-degree relative with a history of venous thromboembolism, hormone replacement therapy, estrogen-containing contraceptive therapy, or varicose veins with phlebitis [7]. However, the prophylactic treatment for thromboembolism has been differently conducted according to the doctor in charge.

We analyzed 8 reports of thromboembolism after operations on upper extremities (Table 1). These cases tended to occur after operation for trauma or for bilateral upper extremities [8-13]. There were 2 patients who had thrombophilia problems before operation [12,14]. One of them had stopped the administration of warfarin and the other had essential thrombocythemia [14]. In addition, the patients with injured or operated limbs required long-term fixation because thromboembolism in veins of the diseased limbs was found after shoulder [10] or remote skin flap operations [13].

**Table 1:** Reported cases of pulmonary embolism after upper extremity operations

**No**	**Author**	**Age**	**Sex**	**Disease**	**Intervention**	**Delay (days)**	**Cause**	**Thrombosis location**
1	Norwood 2004	43	M	L elbow/R forearm Fx	Osteosynthesis	4	?	R iliac vein → PE
2	Starch 2001	73	M	R shoulder cuff tear	Open cuff repair	7	Intraoperative position	Affected upper limb → PE
3	Kim 2012	56	F	B carpal tunnel	B carpal tunnel release	3	Stopping anticoagulants	? → PE
4	Sasaki 2007	19	M	L finger open Fx	Osteosynthesis	17	Obesity	B common iliac vein → PE
5	Takizawa 2006	49	F	L galeazzi Fx	Osteosynthesis	1	?	? → PE
6	Teramoto 2004	85	F	R humerus proximal Fx	Osteosynthesis	4	Essential thrombocytopenia	? → PE
7	Watanabe 2012	58	F	B distal radius Fx	Osteosynthesis	1	Bedrest (preoperative 4 days)	? → PE
8		41	M	L finger injury	Pedicled groin flap surgery	12	Fixed position	L subclavicle vein → PE

M: Male, F: Female, Fx: Fracture, R: Right, L: Left, B: Bilateral ?: Unknown, PE: Pulmonary embolism.

Although our patient experienced bilateral trauma, she did not need fixation, she was allowed to perform range of motion exercises, and she was not confined to specific body positions. This point differs from the reports of Norwood *et al*. and Watanabe *et al*. [8,13]. Moreover, our patient had no previous thrombophilia problems.

Our patient was not motivated to leave her bed after the operation even though she could walk on the operation day. As a result, she remained in bed until the ninth post-operative day. This is the reason why the pulmonary thromboembolism occurred. We believe she had difficulty of leaving her bed because of her age, pain, and anxiety. According to Nickinson *et al*. researchs on the occurrence of anxiety and depression after operation, they reported that elderly patients and women tended to experience depression after operation [15]. Therefore, we should take depression into our consideration strongly after operations in elderly women. The patient mentioned in this report refused to leave her bed and was unwilling to ride in a wheelchair regardless of repeated admonitions from the doctors and nurses.

We believe it was insufficient to manage pain control by using non-steroidal anti-inflammatory drugs. Stronger medications such as opioids used after total knee arthroplasty [16] should have been administered. Insufficient pain control after operation might contribute to depression, therefore, we should consider using opioids under general conditions. Likewise, it is necessary for us to have a cooperation from exparts on depression for those experienced anxiety and depression.

## Conclusion

The patient did not have any “strong” risk factors as reported in the Japanese Orthopedic Association Clinical Practice Guideline on the Prevention of Venous Thromboembolism in Patients Undergoing Orthopedic Treatments. In general, upper extremity operation carries a low risk for pulmonary thromboembolism. For patients with decreased activity of daily living (ADL) and depression after operation, we should consider postponing discharge and performing rehabilitation until their ADL is improved.

## Consent

Written informed consent was obtained from both the patient and her daughter for publication of this case report and accompanying images. A copy of the written consent is available for review by the Editor-in-Chief of this journal.

## Abbreviations

ACCP: American College of Chest Physicians Evidence-Based Clinical Practice Guidelines; JOA: Japanese orthopaedic association; NICE: National Institute of Health and Clinical Excellence Guidelines; AO: Arbeitsgemeinschaft für Osteosynthesefragen; CT: Computed tomography; ADL: Activity of daily living.

## Competing interests

The authors declare that it has no competing interests.

## Author’s contributions

YI (first author) mainly wrote this manuscript and was an assistant of operative procedure. KN (corresponding author) mainly performed medical examinations and surgery for this patient. YS, KK and OO discussed and advised about the treatment for this patient. All authors read and approved the final manuscript.
